# Errata

**DOI:** 10.4269/ajtmh.2011.853err

**Published:** 2011-09-01

In *Am J Trop Med Hyg 85:* 105–112 by Hartung et al, the first and corresponding author's affiliation and current address are incorrect. TK Hartung's affiliation should be listed as Department of Medicine, College of Medicine, University of Malawi, Blantyre, Malawi and Department of Respiratory Medicine, Victoria Hospital, Kirkcaldy, United Kingdom. His current address should be Department of Respiratory Medicine, Victoria Hospital, Hayfield Road, Kirkcaldy, KY2 5AH Kirkcaldy, United Kingdom. E-mail: thomas.hartung@nhs.net.

In *Am J Trop Med Hyg 85:* 303–308 by Jutla et al, the affiliation of some of the authors was incorrectly listed in the print version of the article. The acronym for the Water and Environmental Research, Education and Actionable Solutions Network should have been listed as WE REASoN.

